# The impact of direct oral anticoagulants in traumatic brain injury patients greater than 60-years-old

**DOI:** 10.1186/s13049-018-0487-0

**Published:** 2018-03-27

**Authors:** Oliver Prexl, Martin Bruckbauer, Wolfgang Voelckel, Oliver Grottke, Martin Ponschab, Marc Maegele, Herbert Schöchl

**Affiliations:** 1 Department of Anaesthesiology and Intensive Care Medicine AUVA Trauma Centre Salzburg, Academic Teaching Hospital of the Paracelsus Medical University, Dr. Franz Rehrl Platz 5, 5020 Salzburg, Austria; 2 Paracelsus Medical University, Salzburg, Austria; 3 Department of Anaesthesiology, RWTH Aachen University Hospital, Aachen, Germany; 4 Department of Anaesthesiology and Intensive Care Medicine AUVA Trauma Centre Linz, Academic Teaching Hospital of the Paracelsus Medical University, Salzburg, Austria; 5 Department for Trauma and Orthopaedic Surgery, Cologne-Merheim Medical Centre (CMMC), University Witten/Herdecke (UW/H), Campus Cologne-Merheim, Cologne, Germany; 6 Ludwig Boltzmann Institute for Experimental and Clinical Traumatology, AUVA Trauma Research Centre, Vienna, Austria

**Keywords:** DOAC, Vitamin K antagonist, Brain trauma, Mortality, Intracranial haematoma

## Abstract

**Background:**

Traumatic brain injury (TBI) is the leading cause of death among trauma patients. Patients under antithrombotic therapy (ATT) carry an increased risk for intracranial haematoma (ICH) formation. There is a paucity of data about the role of direct oral anticoagulants (DOACs) among TBI patients.

**Methods:**

In this retrospective study, we investigated all TBI patients ≥60-years-old who were admitted to the intensive care unit (ICU) from January 2014 until May 2017. Patients were grouped into those receiving vitamin K antagonists (VKA), platelet inhibitors (PI), DOACs and no antithrombotic therapy (no-ATT).

**Results:**

One-hundred-eighty-six, predominantly male (52.7%) TBI patients with a median age of 79 years (range: 70–85 years) were enrolled in the study. Glasgow Coma Scale and S-100β were not different among the groups. Patients on VKA and DOACs had a higher Charlson Comorbidity Index compared to the PI group and no-ATT group (*p* = 0.0021). The VKA group received reversal agents significantly more often than the other groups (*p* < 0.0001). Haematoma progression in the follow-up cranial computed tomography (CCT) was lowest in the DOAC group. The number of CCT and surgical interventions were low with no differences between the groups. No relevant differences in ICU and hospital length of stay were observed. Mortality in the VKA group was significantly higher compared to DOAC, PI and no-ATT group (*p* = 0.047).

**Discussion:**

Data from huge registry studies displayed higher efficacy and lower fatal bleeding rates for DOACs compared to VKAs. The current study revealed comparable results. Despite the fact that TBI patients on VKAs received reversal agents more often than patients on DOACs (84.4% vs. 24.2%, *p* < 0.001), mortality rate was significantly higher in the VKA group (*p* = 0.047).

**Conclusion:**

In patients ≥60 years suffering from TBI, anticoagulation with DOACs appears to be safer than with VKA. Anti-thrombotic therapy with VKA resulted in a worse outcome compared to DOACs and PI. Further studies are warranted to confirm this finding.

## Background

Severe traumatic brain injury (TBI) remains the leading cause of death among trauma patients [1]. Multiple studies have identified a peak in TBI incidence among the oldest age groups [2]. Compared to young and previously healthy TBI victims, elderly patients showed worse outcome. This is, in part, linked to age-related comorbidities. The prevalence of cardiovascular and cerebrovascular diseases, such as arterial fibrillation, coronary heart illness or stroke, increases with advanced age. Thus, antithrombotic therapy (ATT) with platelet inhibitors (PI) or vitamin K antagonists (VKA) are often prescribed in the geriatric population. Multiple studies have shown that patients under ATT carry an increased risk for intracranial haemorrhage (ICH) or haematoma progression following severe TBI [3–5].

In recent decades, VKA has been the most frequently prescribed anticoagulant. Direct oral anticoagulants (DOACs) represent a newer class of ATT. Thrombin inhibitors, such as dabigatran and Xa inhibitors (e.g., rivaroxaban, apixaban and edoxaban) block the active site of either thrombin or factor Xa [6]. DOACs show a higher efficacy and lower spontaneous bleeding rate than VKAs [7]. Severe life-threatening and fatal bleeding episodes were significantly lower compared to VKAs in most of these studies [7–10]. Therefore, DOACs are increasingly used and have already matched VKAs prescription in many indications [11].

As a consequence, trauma care providers are faced with an increasing number of injured patients under DOAC medication [12, 13]. Particularly for TBI patients, there remains uncertainty about whether DOACs provoke higher bleeding rates or carry a greater risk for posttraumatic haematoma progression compared to patients on VKAs or PIs [14].

Thus, we performed a retrospective analysis of patients admitted to our level 1 trauma centre suffering from TBI with concomitant intake of DOACs, VKAs or PIs. Patients with no-ATT served as a control group. Our main and secondary targets were mortality and progression of ICH, as well as intensive care unit (ICU) and hospital length of stay.

## Methods

**Fig. 1:**
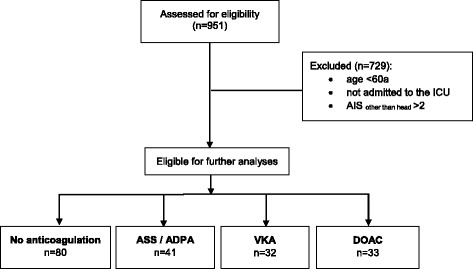
Flowchart of the study. AIS, abbreviated injury score; ASS, aspirin; ADPA, adenosine diphosphate antagonist; VKA, vitamin K antagonist; DOAC, direct oral anticoagulant; ICU, intensive care unit

Following local ethics committee approval (415-E/2228/3-2017), we performed a retrospective analysis of patients admitted with the diagnosis TBI to the AUVA Trauma Centre Salzburg, Austria between January 2014 and May 2017. During the study period 951 patients were admitted to the hospital with the diagnosis “traumatic brain injury”. The majority of these patients suffered from minor brain trauma. TBI patients were eligible for enrolment in the current study if they were ≥ 60-years-old with proven ICH by initial cranial computed tomography (CCT) or considered to be at risk of delayed development of an intracranial bleeding. All patients were transferred from the emergency room (ER) or operation theatre to the ICU. All TBI patients were screened for intake of PIs, VKAs or DOACs. To establish comparable groups, patients were excluded if they were < 60-years-old or transferred from the ER to the normal ward. (Fig. 1).

Patients were stratified into four groups: Group 1 includes patients without antithrombotic therapy (no-ATT), group 2 comprises patients on PIs (e.g., aspirin or adenosine diphosphate antagonists), group 3 consists of patients on VKA (e.g., phenprocoumon or acenocoumarol) and group 4 are patients taking any DOACs (e.g., dabigatran, rivaroxaban, edoxaban and apixaban).

The following data were collected from the electronic anaesthesia and ICU database (COPRA 6): demographics, mechanism of injury, Abbreviated Injury Scale (AIS), Injury Severity Score (ISS) and Glasgow Coma Scale (GCS). Co-morbidities were calculated according to the Charlson Comorbidity index [15].

After the initial CCT, intracranial haemorrhage was reassessed 12–24 h after hospital admission, depending on the patients’ condition and neurologic status. Potential haematoma progression was identified and documented by a radiologist. Finally, we collected information about the use of reversal agents, number of CCT scans, neurosurgical interventions, ICU length of stay, hospital length of stay and mortality.

### Laboratory analyses

Routine laboratory testing at ER admission comprised haemoglobin (Hb), platelet count, prothrombin time index (PTI) and activated partial thromboplastin time (aPTT). Inhibition of platelet aggregation was analysed by Multiplate (Roche Diagnostics, Mannheim, Germany). The following tests are also part of our standard protocol if brain trauma is suspected or intake of PI is reported: arachidonic acid test (ASPI), adenosine-diphosphate test (ADP) and thrombin receptor activating peptide-6 test (TRAP). Protein S-100β, a marker of brain tissue injury, was measured upon ER admission. Finally, the glomerular filtration rate was calculated using the Cockcroft-Gault formula [(140-age) / creatinine (mg/dl)] x [BW (kg) /72] (female × 0.85).

#### Statistics

The normality of the data was tested using the D’Agostino and Pearson omnibus normality test. Continuous variables were expressed as the median and interquartile ranges (25th percentile, 75th percentile). Categorical variables were analysed using χ2. For continuous variables, between-group differences were tested using analysis of variance and Dunnett’s comparison of all columns vs. control column (patients without any antithrombotic therapy). Statistical calculations were performed using GraphPad Prism 5.03 (GraphPad Software, La Jolla, CA, USA). The level of significance was set at *p* < 0.05.

## Results

Between January 2014 and May 2017, 186 predominantly male (52.7%) TBI patients with a median age of 79 years (range: 70–85 years) were admitted from the ER/OR to the ICU for further observation. Eighty patients had no history of ATT intake (any type). Forty-one patients had an ongoing medication with PIs (aspirin [36] and clopidogrel [5]), 32 patients were anticoagulated with VKA (phenprocoumon [13] and acenocoumarol [19]) and the remaining 33 with DOACs (rivaroxaban [21], apixaban [3] and dabigatran [9]). Patients receiving no ATT intake were significantly younger than the patients receiving ATTs (*p* > 0.0001). Patients on VKAs and DOACs had a higher Charlson Comorbidity Index compared to the PI group and no-ATT group (*p* = 0.0006). The demographic and clinical parameters upon ER admission are outlined in Table 1.

**Table 1:** Demographic and clinical data

	no-ATT	PI	VKA	DOAC	*p* value
*n*	80	41	32	33	
Age	74 (64.5–81)	80 (72–86)	81 (74–85)	82 (75.5–84.5)	< 0.0001
Male gender (n/%)	48 (60%)	21 (51.2%)	15 (46.9%)	14 (42.4%)	ns
GCS	14 (12–15)	14.5 (12.75–15)	14 (13–15)	14 (14–15)	ns
AIS Head	3 (2–4)	3 (3–4)	3 (3–4.75)	3 (2.25–4)	ns
ISS	13 (9–17)	14.5 (9–18)	14.5 (9.25–23.75)	10 (9–16)	ns
Charlson Comorbidity Index	3 (2–5)	4 (3.5–5)	4 (4–6)	5 (4–6)	0.0006
Number of CCT	3 (2–5)	3 (3–5)	3.5 (2–6)	3 (2–5)	ns
ICH progression (n/%)	27 (33.7)	16 (39.0)	19 (59.4)	8 (24.2)	0.023
ICU length of stay	46 (21.25–112.3)	47 (25–108)	70 (29.25–159.5)	49 (22–92)	ns
Hospital length of stay	242 (123–407.5)	217 (121–413)	278.5 (109.5–368)	168 (76–345.5)	ns

*no-ATT* no antithrombotic therapy, *PI* platelet inhibitors, *VKA* vitamin K antagonists, *DOAC* direct oral anticoagulants, *GCS* Glasgow coma scale, *AIS* abbreviated injury score, *ISS* injury severity score, *CCT* cranial computer tomography, *ICH* intracranial haematoma, *ICU* intensive care unit, *ns* not significant

ANOVA and Dunnett’s comparison of all columns vs. control column

Chi square test

Median (interquartile range, 25th–75th)

The mechanisms of injury are outlined in Table 2. The most common causes of trauma were low- and high-level falls, followed by bicycle accidents.

**Table 2:** Mechanism of injury

	no-ATT	PI	VKA	DOAC	*p* value
Fall < 1 m	35 (43.8%)	26 (63.4%)	21 (65.6%)	27 (81.8%)	0.0013
Fall > 1 m	17 (21.3%)	9 (22%)	2 (6.3%)	4 (12.1%)	< 0.0001
MVA	8 (10%)	3 (7.3%)	3 (9.4%)	2 (6%)	ns
Bicycle Accident	16 (20%)	2 (4.9%)	2 (6.3%)	0 (0%)	0.0041
Skiing Accident	3 (3.8%)	0 (0%)	3 (9.4%)	0 (0%)	ns
Direct force against head	1 (1.3%)	1 (2.4%)	1 (3.1%)	0 (0%)	ns

*no-ATT* no antithrombotic therapy, *PI* platelet inhibitors, *VKA* vitamin K antagonists, *DOAC* direct oral anticoagulants, *MVA* motor vehicle accident, *ns* not significant

Chi square test

Laboratory data upon ER admission are shown in Table 3. PTI and platelet count were significantly lower and INR significantly higher in patients on VKA compared with the other groups (*p* > 0.0001). The mean INR in the VKA group was 2.6 ± 0.9. Thus, the majority of patients were within the recommended range for sufficient anticoagulation. In the PI group, patients revealed significantly lower values for the ASPI test in the platelet aggregometry analyses compared to VKA and DOAC patients (*p* < 0.0084). From the 23 measurements, 20 revealed effective platelet inhibition.

**Table 3:** Laboratory values upon emergency room admission

	no ATT	PI	VKA	DOAC	*p* value
Hb	13.7 (12.4–14.6)	13.5 (11.5–14.9)	13.3 (11.7–14.2)	12.8 (11.8–13.8)	ns
PTI	104 (95–112)	102 (91–115)	30.5 (23.5–36.0)	70 (56.5–83)	< 0.0001
INR	0.98 (0.93–1.04)	1 (0.93–1.06)	2.51 (2.04–3.24)	1.3 (1.1–1.97)	< 0.0001
aPTT	26.9 (25.1–28.8)	27.9 (26.6–30.2)	39.7 (34.6–41.9)	34.1 (28.8–41.0)	< 0.0001
PLT	228 (182–262)	206 (162.5–257.5)	181 (165–212.5)	216 (172–257)	0.0355
S 100β initial	0.66 (0.28–1.53)	0.34 (0.12–0.95)	0.35 (0.13–1.13)	0.25 (0.12–0.97)	ns
Creatinine	0.88 (0.74–1.02)	0.9 (0.67–1.09)	1.02 (0.8–1.29)	1 (0.8–1.2)	ns
GFR	70.6 (51.5–93.4)	64.18 (48.7–87.7)	56.6 (44.1–72.5)	58.7 (44.3–76.1)	ns
AST	25 (20–39)	22 (17.25–29)	27 (19–34)	25 (17–34.5)	ns
Multiplate®					
ASPI* _(*n* = 16, 23, 6, 5)_	86.5 (42.5–117.8)	19 (11–39)	61 (20–115)	68 (33–125)	0.0084
ADP _(*n* = 16, 11, 6, 5)_	87.5 (62.25–115.3)	50 (33–84)	72.5 (40–135)	86 (66–116)	ns
TRAP _(*n* = 16, 10, 6,5)_	124 (88.5–146.8)	99.5 (93.3–112.3)	109.5 (94.3–128.5)	127 (108.5–152.5)	ns

*No-ATT* no antithrombotic therapy, *PI* platelet inhibitors, *VKA* vitamin K antagonists *DOAC*, direct oral anticoagulants, *Hb* haemoglobin, *PTI* prothrombin time index, *aPTT* activated partial thromboplastin time, *INR* international normalized ratio, *PLT* platelet count, *GFR* glomerular filtration rate, *AST* Aspartate Aminotransferase, *ASPI-test* arachidonic acid test, *ADP-test* adenosine diphosphate test, *TRAP-test* thrombin receptor activated peptide test, *ns* not significant

ANOVA and Dunnett’s comparison of all columns vs. control column

Median (interquartile range, 25th–75th)

S-100β, creatinine, glomerular filtration rate and Aspartate Aminotransferase did not differ significantly between groups.

**Fig. 2:**
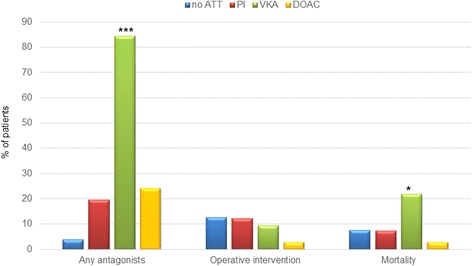
No-ATT versus PI, VKA and DOAC in TBI patients. No-ATT, no antithrombotic therapy; PI, platelet inhibitors; VKA, vitamin K antagonists; DOAC, direct oral anticoagulants; TBI, traumatic brain injury; *p < 0.05; ***p < 0.0001

Patients in the VKA group received a specific antagonist, such as prothrombin complex concentrates (PCC) and vitamin K, significantly more often than the other groups (*p* < 0.0001). In contrast, 3.8% in the non-ATT group, 19.5% in the PI group and 24.2% in the DOAC group received haemostatic therapy. In the DOAC group, four patients on dabigatran were treated with the specific antagonist Idarucizumab, in another four patients the anticoagulant effect of Xa inhibitors was reversed with PCC (Fig. 2).

The median number of CCTs were not different between the groups (Table 1). Haematoma progression in the follow-up CCT was significantly higher in patients on VKAs compared with the remaining groups (*p* = 0.023). The overall number of surgical interventions was low, without differences between the groups (Fig. 2). No relevant differences in ICU and hospital length of stay were observed. In-hospital mortality in the VKA group was significantly higher compared with DOACs, PI groups and non-ATT group (*p* = 0.047) (Fig. 2).

## Discussion

Recent guidelines have recommended DOACs as the first-line anticoagulant therapy for prevention of stroke in non-valvular atrial fibrillation [16]. Moreover, DOACs are increasingly prescribed for the avoidance of thrombo-embolic events in different clinical scenarios. Consequently, trauma care providers are currently facing a growing number of TBI patients on DOACs. Data from huge registry studies revealed higher efficacy and lower spontaneous bleeding rates for DOACs compared to VKAs [8–10, 17]. These findings are, in part, supported by the current study, which shows a significantly lower mortality rate in TBI patients on DOACs compared to VKAs. The results of the current study are of particular interest as patients on VKAs received reversal agents, such as PCC and vitamin K, more often than patients on DOACs (84.4% vs. 24.2%, *p* < 0.001).

Current data suggests that approximately 3–4% of all trauma patients are under ATT before admission [3, 13]. TBI patients with preinjury intake of ATT carry a higher risk for ICH formation, haematoma progression and higher mortality rates compared to those without exposure to these substances [3, 4, 18–20]. However, the risk-benefit ratio of DOACs in TBI patients remains uncertain and has yet to be assessed.

In a mixed subset of 81 anticoagulated trauma patients, Wood et al. reported that DOAC intake resulted in higher survival rates to discharge compared with patients on VKAs (92% vs. 72%, *p* < 0.03) [13]. Interestingly, the rates of ICH were comparable between VKA and DOAC patients (70.0% vs. 66.7%, *p* = 0.74). In a cohort of 247 TBI patients, Pozzessere et al. observed no difference in the incidence of ICH and mortality between VKAs and dabigatran [21]. Myers et al. investigated trauma patients on rivaroxaban and warfarin. No difference for bleeding complications (37% vs. 39%, *p* = 0.49), thromboembolic events (4.2% vs. 5.7%, *p* = 0.44) or mortality (4.2% vs. 5.8%, *p* = 0.63) were detected. Fewer patients under rivaroxaban underwent surgical or interventional radiology procedures (32% vs. 43%, *p* = 0.01) [22]. Maung et al. revealed a non-significant trend towards higher mortality in TBI patients in the warfarin group compared with DOAC patients and those without anticoagulant therapy (19.3% vs. 16.7% vs. 10.9%, *p* = 0.08) [12].

In line with our findings, Feeney et al. also reported a higher mortality in 162 TBI patients with concomitant VKA intake than in DOAC patients. In the VKA group, the need for surgical interventions (26.7% vs. 8.2%, *p* = 0.023) and mortality (20.9% vs. 4.9%, *p* < 0.008) were significantly higher than in the DOAC group [23].

Finally, Kobayashi et al. published the results of a large multicentre observational study from 16 US trauma centres. These authors compared the outcome parameters of 182 TBI patients on DOACs with 605 patients on VKA, 443 on clopidogrel and 478 on aspirin. Mortality was not different between medication groups. The univariate and multivariate analyses did not reveal any higher risk for ICH and progression of intracranial bleeding in patients on DOACs compared to VKAs (19% in each group). Interestingly, this study discovered that aspirin intake was associated with the highest rate of ICH diagnosed upon admission [24].

To improve outcome following TBI, rapid reversal of anticoagulation seems crucial [20, 25]. VKA reversal can be established within minutes by PCC [26–28]. For dabigatran, the specific antibody Idarucizumab is available and allows an immediate inhibition of its anticoagulant effect [29]. Andexanet alpha, a modified decoy for Factor Xa, is currently under development and the first clinical studies have been published recently [30]. However, at the time of writing, this substance has not received approval for clinical use. Thus, only unspecific haemostatic agents, such as PCC or activated PCC are supported by results from preclinical studies and recommended by international guidelines [31]. However, clinical data supporting this therapeutic concept in bleeding patients under Xa inhibitors remain spare [32]. Neither the efficacy nor safety aspects (e.g., potential thromboembolic risk) of these unspecific antagonists have been adequately assessed.

Although all available studies reported much higher rates of pharmacological reversal for patients on VKA compared to DOACs, these treatments were not consistently associated with improved outcome. In our study, the majority (84.4%) of patients on VKA received either PCC or vitamin K, or both. In contrast, only 24.2% of DOAC patients were treated with the specific antagonist Idarucizumab or PCC (*p* = 0.024). This is comparable to the report of Wood et al., where 60% of patients on VKA and only 19% of DOAC patients received reversal agents [13]. In a multicentre study published by Kobayashi et al., the anticoagulant effect of warfarin was reversed in only 47% compared with 13% of the DOAC patients (*p* < 0.001) [24]. Barletta et al. reported similar rates of pharmacological reversal (48.1% [warfarin] vs. 13.8% [DOAC], *p* > 0.001) but, despite this, mortality was comparable between warfarin and DOAC patients (5.9% vs. 4.3%, *p* = 0.789) [33]. Myers et al. also reported that fewer patients under rivaroxaban received reversal agents than patients under VKA (20% vs. 29%, *p* = 0.02) with no effect on bleeding complications [22].

INR is the established gold standard to rapidly measure the effect of VKA; whereas laboratory diagnosis is more complex for patients on DOACs. Standard coagulation tests are not sensitive enough for assessment their inhibition on the coagulation process [34]. Specific tests, which allow quantification of the concentration of Xa inhibitors or dabigatran, are not universally available in all hospitals. This fact might also influence the low number of DOAC patients who received a reversal agent in our study, as well as in all so far published studies. Due to the increasing number of DOAC patients admitted to our trauma hospital, we recently established these assays in our laboratory to provide rapid information about the anticoagulant status of these patients.

### Limitations

Some limitations of the current study should be noted. The overall sample size is relatively small. Therefore, we cannot rule out that patients on VKA suffered from more severe TBI compared to patients on DOACs. However, we found no differences in S-100β and GCS, suggestive of comparable brain trauma severity. Moreover, the Charlson Comorbidity Index was also similar between groups. The DOAC group consists of both factor Xa inhibitors and direct thrombin inhibitors. Due to the small sample size, we were unable to investigate relevant outcome differences between Xa and thrombin inhibitors.

According to the INR, the majority of patients on VKAs were in the therapeutic range. Aggregometry analyses of patients on PIs also showed relevant inhibition of platelet function in the majority of measurements. As we did not quantify the concentration of Xa inhibitors or dabigatran on a regular basis, we cannot report whether the level of anticoagulation was comparable between VKA and DOACs.

In contrast to TBI patients on VKA, we did not establish a standardised protocol for administration of reversal agents in DOAC patients. However, since the approval of Idarucizumab, this agent is our standard first-line therapy in TBI patients on dabigatran.

## Conclusion

Although TBI patients anticoagulated with DOACs share demographic and clinical similarities with those on VKAs, their mortality was significantly lower. This is of particular interest as significantly more patients on VKAs received reversal agents compared DOAC patients. Our findings are in line with previous results that also showed superior overall outcome in trauma patients on DOACs compared to patients on VKAs.

## Abbreviations

ADP: Adenosine-diphosphate test
AIS: Abbreviated Injury Scale
aPTT: activated partial thromboplastin time
ASPI: Arachidonic acid test
ASS: Aspirin
ATT: Antithrombotic therapy
CCT: Cranial computed tomography
DOAC: Direct oral anticoagulant
ER: Emergency room
GCS: Glasgow Coma Scale
ICH: Intracranial haematoma
ICU: Intensive care unit
ISS: Injury Severity Score
no-ATT: no antithrombotic therapy
PCC: Prothrombin complex concentrate
PI: Platelet inhibitors
PTI: Prothrombin time index
TRAP: Thrombin receptor activating peptide-6
VKA: Vitamin K antagonist
